# EHMTI-0262. Dysregulation of inflammatory pathways in a familial hemiplegic migraine 1 mouse model after the induction of cortical spreading depression

**DOI:** 10.1186/1129-2377-15-S1-B5

**Published:** 2014-09-18

**Authors:** B de Vries, EE Eising, R Shyti, LS Vijfhuizen, LAM Broos, PAC 't Hoen, MD Ferrari, EA Tolner, AMJM van den Maagdenberg

**Affiliations:** 1 Human Genetics, Leiden University Medical Center (LUMC), Leiden, Netherlands; 2 Neurology, Leiden University Medical Center (LUMC), Leiden, Netherlands

## Background

Familial Hemiplegic Migraine type 1 (FHM1) is a rare monogenic subtype of migraine with aura caused by mutations in the CACNA1A gene. In FHM1 knock-in mouse models these mutations increase the susceptibility for cortical spreading depression (CSD): the underlying mechanism of the migraine aura.

## Aim

To study the consequences of CSD in a migraine-relevant context, we measured cortical gene expression profiles in FHM1 and wild-type mice 24 hours after CSD induction.

## Method

Expression profiles were generated using deep-Serial Analysis of Gene Expression (SAGE) sequencing, a tag-based next-generation sequencing method for gene expression profiling. Relevant expression changes were validated by qPCR experiments.

## Results

Our data show that CSD induces differential expression of genes involved in inflammatory pathways in both the FHM1 and wild-type mice. However, we identified a gene set that is up-regulated upon CSD specifically in the FHM1 migraine mouse model. Genes from this gene set are involved in inflammatory and interferon-related signaling, and were often found up-regulated in immune-stimulated conditions.

## Conclusion

Differential expression of genes involved in inflammatory pathways in the brain of FHM1 migraine mice compared to wild-type mice upon CSD, indicates that CSD affects the brain differently in a genetically predisposed animal which may help increase our understanding of migraine pathophysiology.

No conflict of interest

